# Specific insomnia symptoms and self-efficacy explain CPAP compliance in a sample of OSAS patients

**DOI:** 10.1371/journal.pone.0195343

**Published:** 2018-04-04

**Authors:** Pierre Philip, Stéphanie Bioulac, Elemarije Altena, Charles M. Morin, Imad Ghorayeb, Olivier Coste, Pierre-Jean Monteyrol, Jean-Arthur Micoulaud-Franchi

**Affiliations:** 1 Clinique du Sommeil, Service d’Explorations Fonctionnelles du Système Nerveux, CHU de Bordeaux, Bordeaux, France; 2 Univ. Bordeaux, SANPSY, Bordeaux, France; 3 CNRS, SANPSY, Bordeaux, France; 4 Université Laval, 2325, rue de l’Université, QC G1V 0A6 Québec, Canada et Centre de recherche CERVO, Institut universitaire en santé mentale de Québec, Québec, Canada; 5 CNRS, Institut de Neurosciences Cognitives et Intégratives d’Aquitaine, Bordeaux, France; 6 Université de Bordeaux, Institut de Neurosciences Cognitives et Intégratives d’Aquitaine, Bordeaux, France; University of Rome Tor Vergata, ITALY

## Abstract

This study explores the association between specific insomnia symptoms (sleep onset, sleep maintenance and early morning awakenings symptoms) and self-efficacy (perceived self-confidence in the ability to use CPAP) with CPAP compliance in French patients with obstructive sleep apnea syndrome (OSAS). We performed a retrospective, cross-sectional analysis of CPAP compliance in a cohort of 404 patients diagnosed with OSAS. Patients completed mailed questionnaires on sleepiness (ESS), insomnia (ISI) and self-efficacy in sleep apnea (SEMSA). Linear regression modeling analyses were performed to explore the impact of measured variables on the number of hours of CPAP use. Of the initial pool of 404 patients, 288 returned the questionnaires (71% response rate). Their mean age was 63.16±12.73 yrs, 31% were females, mean BMI was 30.39±6.31 kg/m2, mean daily CPAP use was 6.19±2.03 h, mean number of years of use was 6.58±6.03 yrs, and mean initial AHI before CPAP use was 34.61±20.71 /h. Age (p<0.01), BMI (p<0.01), sleep onset insomnia symptoms (p<0.01), sleep maintenance insomnia symptoms (p<0.01) and self-efficacy (p<0.01) were significantly associated with mean daily CPAP use. We found that specific insomnia symptoms and self-efficacy were associated with CPAP compliance. Our findings underline the need to demonstrate that interventions that reduce insomnia symptoms and improve self-efficacy will increase CPAP compliance.

## Introduction

The co-occurrence of obstructive sleep apnea syndrome (OSAS) and insomniac symptoms was first described by Guilleminault et al. in a seminal article [1]. Since then several other studies have highlighted this co-occurrence [2, 3]. In an emblematic and large cohort of 822 patients with OSAS, Björnsdóttir et al. (2013) demonstrated that 68% of patients complained of insomnia before initiating CPAP treatment [4]. Comorbid insomnia and OSAS has been called COMISA to highlight the close bidirectional relationship between these two syndromes [5]. The prevalence of COMISA is variable between studies depending on the population studied and the methods used to evaluate insomnia, especially the type of scale used [5]. In a review by Luyster et al, (2010) the prevalence of COMISA in patients consulting for suspected OSAS was estimated at 39% to 58% [3].

Krakow et al. were first to suggest that patients with COMISA could be less compliant with CPAP treatment [6]. Insomnia symptoms could indeed keep patients with CPAP awake, which in turn could exacerbate the psychophysiological processes of insomnia [5]. In 2008, the first clinical description of the impact of COMISA on CPAP compliance was described in a case study [7]. The study suggested the importance of taking insomnia symptoms into account in order to improve compliance with CPAP. The relationship between COMISA and poor compliance has since been confirmed by six prospective studies [4, 8–12] and two retrospective studies [13, 14]; only one study did not confirm this association [15].

The first study in 2006 showed that 39 patients with sleep onset insomnia (SOI) at baseline and without excessive daytime sleepiness (EDS) exhibited a lower acceptance rate (as evaluated by the rate of devices purchased) than 90 patients with SOI and EDS, and 64 patients with only EDS [11]. The second study in 2009 evaluated insomnia symptoms at baseline with the Insomnia Severity Index (ISI) in 52 patients with OSA and compliance of CPAP use at one year (number of hours of CPAP use per night). Patients with the most severe perceived insomnia (23% of the sample) showed the lowest CPAP compliance after one year as compared to the other groups [12].

Four prospective studies used respectively at baseline a non-validated questionnaire [10], the Regensburg Insomnia Scale (RIS) [9], the ISI [8] and the three dedicated questions of the Basic Nordic Sleep Questionnaire (BNSQ) [4], in respectively, 232, 73, 65 and 705 patients with OSAS. Among these four studies, two addressed the impact of the type of insomnia on CPAP treatment [4, 10]. Severity of insomnia symptoms evaluated with the RIS showed a significant negative association with the number of hours of CPAP use per night six months after initiation [9]. Severity of insomnia symptoms evaluated with the ISI was also a predictor of compliance (CPAP use ≥ 4h / night or CPAP use < 4 h / night) at one week after initiation but not at one month [8]. The association between insomnia severity (as measured by ISI score) and CPAP compliance was confirmed in two retrospective studies in respectively 248 and 207 patients with OSAS conducted by the same team [13, 14].

Interestingly, not only the severity of insomnia symptoms but also the type of insomnia (sleep onset insomnia, sleep maintenance insomnia and early morning awakenings) play a differential role on CPAP compliance. Sleep maintenance insomnia was significantly negatively associated with the number of hours of CPAP use per night one month after the initiation in one study [10]. In the Icelandic sleep cohort [4] patients who reported sleep onset insomnia at baseline on the BNSQ were more likely to be CPAP non-users at follow-up, an effect that remained significant after adjusting for age, sex, BMI, smoking and OSAS severity [4].

Only one study using the ISI evaluation at baseline in 166 patients with OSAS did not find a significant relationship between insomnia symptoms and compliance 6 months after initiation [15]. However, it was suggested that this negative result could be related to the large number of patients consuming hypnotic medications in the French cohort study [5, 15].

To our knowledge, only two retrospective and cross-sectional studies have evaluated the relationship between insomnia symptoms and CPAP compliance in patients treated with OSAS [13, 14]. However, it is important to evaluate insomnia symptoms after CPAP initiation in order to study the relationship between insomnia symptoms and long-term CPAP compliance. Moreover, if CPAP can reduce insomnia symptoms, it is also important to identify which subtype of insomnia is associated with CPAP compliance and if other factors can explain long-term CPAP compliance in a global model. The studies by Wallace et al. did not investigate CPAP compliance in relation to insomnia subtype but other factors were found to affect CPAP compliance: demographic factors (sex, age, BMI), OSAS severity (Apnea and Hypopnea Index), daytime sleepiness and perceived self-confidence in the ability to use CPAP, also called “self-efficacy”[16]. Self-efficacy can be evaluated with a dedicated self-report questionnaire called SEMSA (Self-Efficacy in Sleep Apnea) [17], standard clinical instrument often used to evaluate psychological factors related to OSAS and CPAP. Indeed, the impact of self-efficacy on CPAP compliance as evaluated by the SEMSA has been confirmed in several studies [8, 13, 14, 16, 18–22].

Self-efficacy can thus be considered as a strong predictor of CPAP compliance [16] but the impact of both insomnia symptoms and self-efficacy on CPAP compliance has received little attention [8, 13, 14]. The studies by Wallace et al. (2013, 2015) were conducted in US veterans with OSAS who attended a CPAP follow-up clinic. As this group of participants is not representative of the general population, the results of the study may not apply to groups of participants recruited from the general population. In particular, it is important to investigate the French population in view of the contradictory result obtained in a French sample by Nguyen et al. (2010). Moreover, France is known for the quality and universality of its health care system [23]. Public health insurance covers the entire population and major fees for CPAP while physicians in sleep medicine can choose the type of CPAP equipment (type of machine, type of mask, humidifier etc…) best suited to their patients [24]. Consequently, the French population has a much higher rate of continuous usage of CPAP than the US one. Thus, it is important to investigate previously established variables (demographic, OSAS severity, daytime sleepiness, insomnia symptoms, and self-efficacy) associated with CPAP compliance in a French population. To address this issue, we conducted a retrospective cross-sectional study in a large population of French patients treated by CPAP and followed up in our sleep clinic for several months to predict the best predictive factors associated with CPAP compliance. We hypothesize that insomnia symptoms and self-efficacy will be associated with CPAP compliance.

## Methods and materials

### Participants

The study design was retrospective and cross-sectional. Patients were selected from a cohort of 404 consecutive subjects diagnosed with OSAS according to AASM criteria with polygraphy or polysomnography in the sleep clinic at Bordeaux University Hospital who were then treated by continuous positive airway pressure (CPAP) and followed by the Vitalaire home care provider (see details below under ‘Procedure’). These patients received a letter describing the purpose of the study and inviting them to self-administer a confidential survey. A mailing was conducted in November 2016.

### Procedure

Routine evaluation was performed in the entire cohort of subjects: data on age, sex, BMI, average number of hours of CPAP use per night for the previous month and number of years of CPAP installation were collected by the home care company. After providing written informed consent, the patients filled in a self-report survey and send it to the sleep clinic in a postage-paid envelope. The survey contained the French version of the Insomnia Severity Index (ISI), the French version of the Epworth Sleepiness Scale (ESS) and the Self-Efficacy Measure of Sleep Apnea (SEMSA). In addition, the initial Apnea and Hypopnea Index (AHI) recorded by polygraphy or polysomnography were collected by the sleep clinic. According to the Article L1121-1, LOI n°2011–2012 du 29 décembre 2011—art. 5, ethical approval is not needed for researches in which all actions are performed and products used routinely. This study was conducted in accordance with the Declaration of Helsinki and French Good Clinical Practices.

### Questionnaires

#### ISI

The French validated version of the ISI was used in this study [25, 26]. The ISI is a self-report questionnaire comprised of 7 items rated from 0 to 4 on a 5-point Likert scale. The total scores range from 0 to 28. Higher scores indicate greater severity of insomnia symptoms. Total score categories are classically subdivided as follows: 0–7 = No clinically significant insomnia, 8–14 = Subthreshold insomnia, 15–21 = Clinical insomnia (moderate severity), 22–28 = Clinical insomnia (severe). The first three items of the ISI evaluate symptoms of “Difficulty falling asleep”, of “Difficulty staying asleep” and of “Problems waking up too early”. Each item was scored from 0–4 (none, mild, moderate, severe, very severe). The remaining items evaluate satisfaction with current sleep, degree of daytime impairments, distress, and notice ability of impairments caused by insomnia.

#### ESS

The French validated version of the ESS was used in this study [26, 27]. The ESS is a self-report questionnaire containing 8 items rated from 0 to 3 on a 4-point Likert scale. The total scores range from 0 to 24. Higher scores indicate greater propensity to fall asleep in different day- and nighttime situations. Total score categories are classically subdivided as follows: 0–10 = No clinically significant sleepiness, 11–15 = Excessive daytime sleepiness, 16–24 = Severe excessive daytime sleepiness.

#### SEMSA

Our team recently validated the French version of the SEMSA which we used in this study [28]. The SEMSA is a US-designed self-report questionnaire comprising 26 items rated from 1 to 4 on a 4-point Likert scale [17]. The questionnaire consists of three factors: risk perception of OSAS, benefit (outcome expectancy) of CPAP and self-efficacy in use of CPAP in line with Bandura’s social cognitive theory [17]. The arithmetic mean of the Likert rating for each participant was computed for the overall SEMSA score and for each of the three factors. The total score ranges from 1 to 4. Higher scores indicate greater risk perception, higher benefit expectancy with treatment and greater perceived self-efficacy [17]. The French version of the SEMSA exhibited satisfactory internal consistency and concurrent validity. Moreover, the 3-factor structure was confirmed by a confirmatory factor analysis.

### CPAP treatment

#### CPAP initiation and follow-up

CPAP treatment was begun at home by a certified sleep respiratory technologist from the Vitalaire home care company. The technologist spent a standardized 1-hour session to install the CPAP unit, to adjust the mask and to give information on OSAS, CPAP treatment, the medical consequences of OSAS and the side-effects of CPAP. CPAP was on auto-titration or fixed pression mode according to the sleep prescription. The technologist returned to the participant’s home after 1 month, then annually. Patients were encouraged to contact the technologist if they encountered ongoing difficulties, and the technologist visited the patient when necessary to optimize their care. 3–6 months after treatment initiation, patients were followed up by a consultation with a sleep medicine physician at the sleep clinic, then annually. If intercurrent problems occurred during treatment (nasal obstruction, upper airway infection, concomitant sleep disorders…), patients were seen at the sleep clinic to evaluate the impact on CPAP tolerance.

#### CPAP compliance

All CPAP units contained software that measured and recorded CPAP mask-on time. Compliance measured included the average number of hours of CPAP use per night in the month preceding the assessment by questionnaires sent by mailed survey. Mean residual AHI was also collected.

### Statistical analyses and hypotheses

Descriptive statistics of the data included frequencies and percentages of categorical variables together with means and standard deviations of continuous variables. Comparison of age, sex, BMI, number of years since CPAP treatment initiation, mean daily CPAP use in patients who did not fill in the questionnaire and in those who did was conducted with a Chi2 test for categorical variables and a t-test for continuous variables. Data analysis was performed using SPSS software (Version 18 for Mac, PASW Statistics). For all tests, significance level was 5%.

#### Univariate analysis

Univariate analysis was performed to evaluate the relationship between mean daily CPAP use and ISI scores (total and “Difficulty falling asleep”, “Difficulty staying asleep” and “Problems waking up too early”), ESS scores (initial and post), SEMSA total scores, SEMSA risk perception score, SEMSA outcome expectancy scores, SEMSA self-efficacy score, age, sex, BMI, number of years of CPAP installation, residual IAH and initial AHI. Pearson’s coefficients were obtained for all these variables except sex and initial excessive daytime sleepiness (ESS>10), for which a t-test was performed.

#### Multivariate analysis

Multivariate analysis was performed using linear regression modeling with mean daily CPAP use as explanatory variable and variables found to be significantly different in the univariate analysis. Concerning the ISI score, a series of linear regression analyses was performed to evaluate the relationships for each ISI score (total and “Difficulty falling asleep”, “Difficulty staying asleep” and “Problems waking up too early”).

## Results

### Sample characteristics

The mean age of the 404 patients was 61.59±13.82 yr, 36.6% (148) were females, the mean BMI was 30.86±6,34 kg/m2, the mean daily CPAP use was 6.07±2.31 h and the mean number of years of installation was 6.01±5.89 yrs. Two hundred eighty-eight subjects returned the questionnaire (71% response rate). Their mean age was 63.16±12.73 yr (range 24–90 yr), 31% (91) were females, their mean BMI was 30.39±6.31 kg/m2, and mean daily CPAP use was 6.19±2.03 h. Mean daily CPAP use was not significantly different between males and females (t = -1.28, p = 0.2). 16% of the patients used it <4 h / night, 24% <5 h / night, 40% <6 h / night, and 58% <7 h / night, and the mean number of year of use was 6.58±6.03 yr (<1 yr: 19.7% of the subjects, 1–2 yr: 17.5%, 2-3yrs 12.3%, 3–5 yr: 14.8%, 5–10 yr: 13.3%, and > 10 yr: 22.4%).

**Fig 1:**
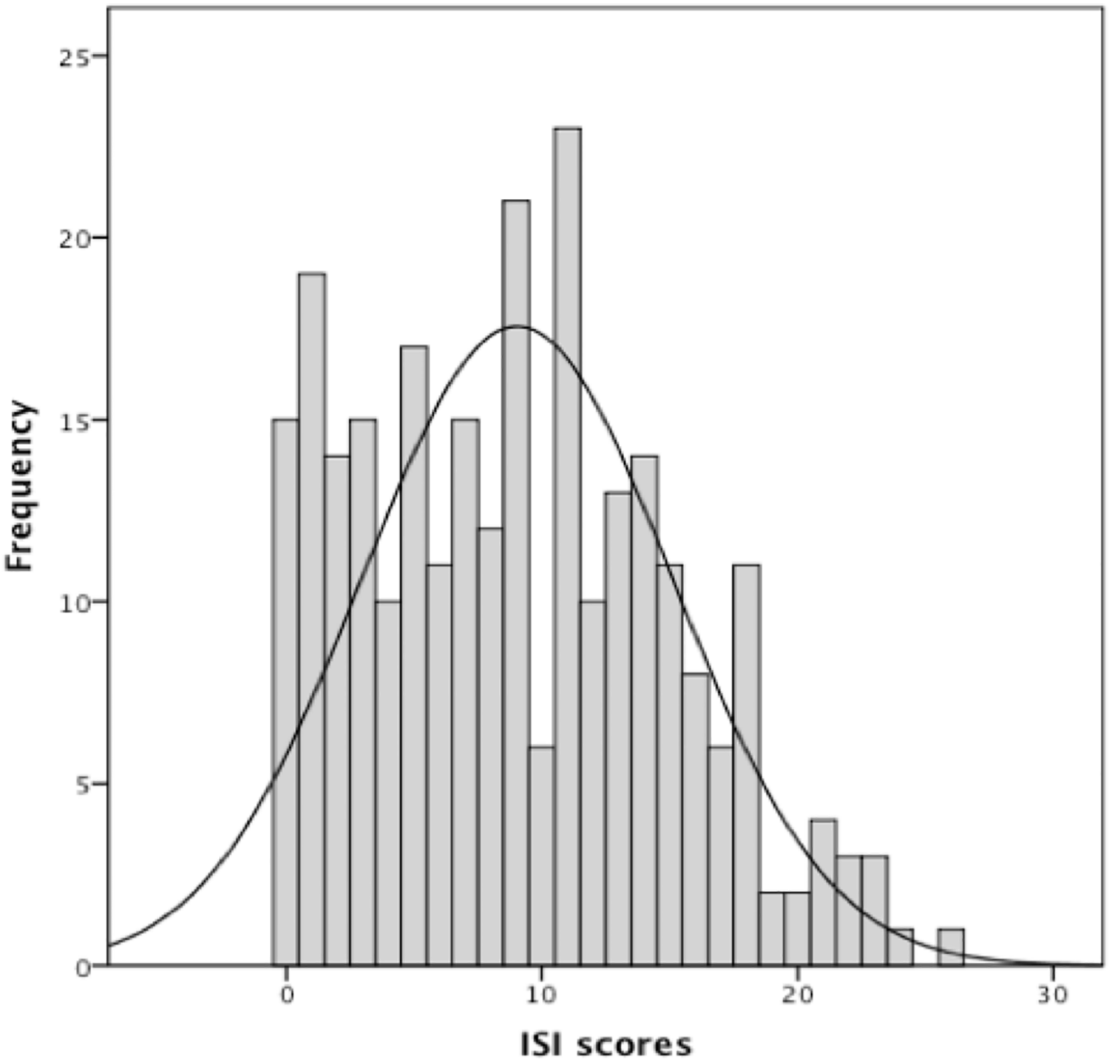
Distribution of ISI scores.

The patients who completed the questionnaire were older (63.16 yr. vs 58.64 yr.) and exhibited a higher mean daily CPAP use (6.19 hr. vs 5.71 hr.) and higher mean number of years of installation (6.58 yr. vs 4.87 yr.) than those who did not. In the 288 subjects, the mean initial ESS score was 9.55±6.07 and the mean initial AHI before CPAP treatment was 34.61±20.71 /h. 47.1% of subjects experienced excessive daytime sleepiness (ESS>10) and 15.1% severe excessive daytime sleepiness (ESS>15). The mean ESS score after CPAP treatment was 4.82±4.01 and the mean residual AHI after treatment was 1.93±2.61 /h. 13.4% of subjects experienced excessive daytime sleepiness (ESS>10) and 1.9% severe excessive daytime sleepiness (ESS>15). The mean ISI score was 9.03±6.06; 19.5% of subjects reported moderate-to-severe insomnia (Fig 1), the mean SEMSA total scores was 2.97±0.46, the mean SEMSA risk perception score was 2.47±0.69, the mean SEMSA outcome expectancy score was 3.22±0.61 and the mean SEMSA self-efficacy score was 3.16±0.67.

### Univariate analysis

Mean daily CPAP use was significantly positively correlated with age (r = 0.26, p = 0.0001), BMI (r = 0.29, p = 0.0001), initial AHI (r = 0.14, p = 0.04), number of years of installation (r = 0.26, p = 0.0001), SEMSA total scores (r = 0.14, p = 0.04) and SEMSA self-efficacy (r = 0.26, p = 0.0001), and negatively correlated with total ISI scores (r = -0.28, p = 0.0001), “Difficulty falling asleep” ISI score (r = -0.22, p = 0.0001), “Difficulty staying asleep” ISI score (r = -0.25, p = 0.0001), “Problems waking up too early” ISI score (r = -0.18, p = 0.002). The ISI score alone explained 7.9% of the variance in mean daily CPAP use. The SEMSA self-efficacy score alone explained 6.6% of the variance in mean daily CPAP use. Mean daily CPAP use was not significantly correlated with residual AHI (r = 0.04, p = 0.47), SEMSA risk perception score (r = -0.019, p = 0.76), SEMSA outcome expectancy scores (r = 0.11, p = 0.075), initial EES score (r = -0.065, p = 0.31) or post ESS score (r = -0.089, p = 0.14). Moreover, it was not significantly different between males and females (t = -1.28, p = 0.2) or between patients with OSA with initial excessive daytime sleepiness (ESS>10) and patients with OSAS without initial excessive daytime sleepiness (t = -.33, p = 0.7).

### Multivariate analysis

Linear regression analysis was performed by including variables determined to be statistically significant in univariate analysis. Owing to the high collinearity between SEMSA total scores and SEMSA self-efficacy, only the SEMSA self-efficacy variable was included in the model. The model containing 6 variables (age, BMI, initial AHI, number of years of CPAP installation, ISI scores and SEMSA self-efficacy score) was significantly associated with mean daily CPAP use and explained around 19% of its variance for each model (Table 1). Model 1 was conducted with total ISI scores, model 2 with “Difficulty falling asleep” ISI score, model 3 with “Difficulty staying asleep” score and model 4 with “Problems waking up too early” score (Table 1).

**Table 1:** Regression analysis of measures associated with mean daily CPAP use.

	b measures	SE b	Beta	P value
**Model 1**				
BMI	0.074	0.018	0.229	**<0.001**
SEMSA self-efficacy score	0.581	0.174	0.181	**<0.001**
**ISI total score**	-0.053	0.019	-0.153	**<0.001**
Age	0.024	0.009	0.150	**0.01**
Number of years of installation of CPAP	0.028	0.020	0.082	0.16
Initial AHI	0.003	0.006	0.026	0.63
**Model 2**				
BMI	0.074	0.018	0.230	**<0.001**
SEMSA self-efficacy score	0.068	0.019	0.230	**<0.001**
**“Difficulty falling asleep” ISI score**	-0.28	0.097	-0.155	0.004
Age	0.028	0.009	0.174	0.003
Number of years of installation of CPAP	0.032	0.02	0.092	0.11
Initial AHI	0.003	0.006	0.029	0.601
**Model 3**				
BMI	0.075	0.018	0.234	**<0.001**
SEMSA self-efficacy score	0.065	0.019	0.182	0.001
**“Difficulty staying asleep” ISI score**	-0.2	0.098	-0.115	0.001
Age	0.026	0.009	0.157	0.007
Number of years of installation of CPAP	0.031	0.02	0.089	0.129
Initial AHI	0.003	0.006	0.024	0.665
**Model 4**				
BMI	0.079	0.018	0.224	**<0.001**
SEMSA self-efficacy score	0.066	0.02	0.184	0.001
**“Problems waking up too early” ISI score**	-0.162	0.093	-0.095	0.084
Age	0.026	0.009	0.162	0.005
Number of years of installation of CPAP	0.034	0.02	0.099	0.091
Initial AHI	0.003	0.006	0.026	0.639

BMI: Body Mass Index, AHI: Apnea Hypopnea Index, CPAP: Continuous Positive Airway Pressure, ISI: Insomnia Severity Index, SEMSA: Self-Efficacy Measure of Sleep Apnea, b: unstandardized regression coefficient, SE: Standard error, Beta: standardized regression coefficient.

## Discussion

This is the first retrospective cross-sectional evaluation in a French population of the factors influencing long-term compliance with CPAP treatment. Insomnia symptoms and self-efficacy have each previously been reported to be important factors associated with CPAP compliance, but only one study has examined both of these factors together and that study was conducted in a selected sample of US veterans, so the results could not be extrapolated at the whole-population level [8, 13, 14]. The present findings confirm that not only insomnia symptoms and self-efficacy but also age and BMI are associated with CPAP treatment compliance. This relationship was adjusted for number of years of installation and OSAS severity.

Our population comprised 288 subjects, which is quite similar to the two previous retrospective studies which included 248 and 207 patients, respectively [13, 14]. However, our population was quite different from these previous populations since there was a more balanced male/female ratio (69%/31% versus around 90%/10% male/female), a much higher mean daily CPAP use (6.1 h / night versus 3.2 h / night), many more years of installation (6.58 yr. versus 1.4 yr.), a lower level of daytime sleepiness (mean ESS 4.8 versus 10.1) and a lower rate of insomnia symptoms (19.5% versus 45% moderate-to-severe insomnia). Interestingly, SEMSA score, age, BMI, and OSAS severity were quite similar. Our study thus shows that in a sex-balanced population of regular and longtime users of CPAP treatment, severity of insomnia symptoms and self-efficacy are key factors affecting CPAP compliance.

Unlike the French study of Nguyen et al. [15] but like the majority of other studies [4, 8–14], we found that insomnia symptoms and its severity are related to CPAP compliance. Importantly, insomnia symptoms were evaluated after CPAP initiation in our study. The insomnia symptoms can be considered therefore to be not reduced by CPAP initiation, nor were they the result of the first days of treatment with it. They were therefore symptoms that could have affected long-term CPAP compliance. The present results thus confirm the need to assess insomnia symptoms not only before CPAP initiation [5] but also in the follow-up of patients treated with CPAP for OSAS [3]. This also suggests that it may be necessary to implement insomnia-specific treatment even in the context of OSA. Moreover, if insomnia symptoms at baseline are associated with future poor compliance with CPAP [4], CPAP treatment may in turn reduce insomnia symptoms in specific patients. By treating repeated episodes of apnea, the associated sleep fragmentation is also reduced, which can improve sleep quality and reduce insomnia symptoms [5]. The same French research team who did not find any impact of insomnia on future CPAP compliance [15] found in 73 patients with COMISA that around 25% of them exhibited a clinically significant improvement of the severity of their insomnia symptoms on the ISI two years after treatment initiation [29]. This result was confirmed in a cohort of 53 patients with COMISA from the United States with the first three items of the ISI [30]. That study showed that patients with more severe OSAS at baseline (high respiratory disturbance index) are more likely to exhibit an improvement of their insomnia symptoms [30]. Moreover, the results of the three dedicated questions of the BNSQ in the Icelandic sleep cohort showed that patients with sleep maintenance insomnia at baseline were more susceptible to a decrease in their insomnia symptoms and to continue using CPAP two years after treatment initiation [4].

Our results show that sleep onset insomnia symptoms and sleep maintenance insomnia symptoms are related to CPAP compliance, early morning awakenings are not, findings that are in line with previous studies [4, 10, 11]. This could be explained by the fact that most patients with OSAS tend to use their treatment less in the second part of the night at a time when sleep pressure is lower [31], without experiencing symptoms of insomnia. Thus, patients treated with CPAP could be more likely to stop their CPAP treatment in their sleep in the early morning [4].

We also found that self-efficacy is a key element related to treatment compliance. Patients confident in their ability to use CPAP may be more likely to tolerate and use it at night. Interestingly, this is the first time that a US-designed scale (SEMSA) [17] has identified similar behavior in a very different medico-social model. Indeed, healthcare is free in France and financial issues account for only a small part of the motivation patients have when seeking treatment [23]. Moreover, alternatives therapies like oral appliances are also reimbursed so long-term CPAP compliance, unlike in other western countries, is only slightly affected by financial issues [24].

The other factors independently associated with CPAP compliance were age and BMI. Age was already found to be a predictor of CPAP compliance in previous studies, probably owing to the better tolerance to chronic treatment [32]. BMI is also a factor already found in previous studies [33] and one may suspect that overweight patients show more severe symptoms and therefore benefit more from treatment. This hypothesis is nevertheless questionable because initial AHI is not predictive of better CPAP compliance. High BMI is possibly correlated with higher levels of nocturnal sleepiness [34], which could explain the better tolerance. Quite surprisingly, years of CPAP use did not explain CPAP compliance, perhaps because the benefits of CPAP vary in terms of hours of use among patients and because some very good responders have used CPAP only a few hours per night for many years [16].

The present study has several limitations. First, it did not assess socio-economic status, education level and marital status which are known to be related not only to CPAP compliance [16] but also to self-efficacy [35] and insomnia symptoms [36]. Further research is thus needed to investigate the impact of such variables on the relationship between self-efficacy and insomnia symptoms, and CPAP compliance. Second, several biomedical factors were not investigated despite their impacts on CPAP compliance: hypnotic consumption [5, 15], smoking status [37], hypertension at baseline [38], cardiovascular comorbidies [39] and psychiatric cormorbidities [40]. In the study by Nguyen et al., it was suggested that the large number of patients consuming hypnotic medications in France could explain the absence of relationship between insomnia symptoms and CPAP compliance [5, 15]. Thus, further research is needed to investigate the effect of hypnotic medication, but also other drugs often prescribed for insomnia symptoms, on CPAP compliance [41]. The smoking status is also independently related to compliance to CPAP therapy [37]. Moreover, it has been reported that current smokers were more likely to have insomnia symptoms [42, 43], Thus, further research should investigate the impact of tobacco consumption in the relationship between insomnia symptoms and CPAP compliance. Hypertension in patients treated with OSA without sleepiness is associated with CPAP compliance. Thus, hypertension should be also investigated in further research. Moreover, cardiovascular [39] and psychiatric [40] comorbidities can also have an impact on CPAP compliance. These comorbidities are related to a high degree of biomedical phenotype heterogeneity in patients with OSA [44, 45]. Thus, further studies should investigate the relationship between these comorbidities and insomnia complaints as determinants of CPAP compliance. Third, only the average number of hours of CPAP use per night was investigated, while information about factors influencing compliance to CPAP can be lost by averaging [13, 46]. Thus, further studies are needed to investigate not only mean daily CPAP use but also the impact of insomnia symptoms and self-efficacy on the percentage of nights used or percentage of nights > 4 h. Fourth, our results report the effects only of patients who agreed to respond to the survey. Thus, the results may have been partly biased by the fact that the most engaged patients to this issue have responded. Nevertheless, the response rate was very high and despite significant differences in age, mean daily CPAP use and mean number of years of installation, the differences can be considered as clinically limited. Fifth, the study sample consisted of patients followed by a home-care service and returning to our sleep-clinic, so no data were available on patients who stopped the treatment and for whom CPAP was discontinued. Moreover, this study is limited by its cross-sectional design instead of prospective design. No causal inference can formally be made. Prospective studies are thus required to confirm these results and to investigate the causal inference between insomnia symptoms and CPAP compliance.

Despite these limitations, our results highlight the need in patients with specific insomnia symptoms to look carefully for CPAP compliance throughout the period of treatment. Moreover, France is now adopting a new reimbursement system for CPAP, which will be conditioned by the hours of CPAP use. Education is a key factor and the impact of self-efficacy on CPAP use should reinforce educational programs in at-risk populations [47]. Sleep hygiene and cognitive behavioral therapy for insomnia help in reducing insomnia symptoms [48, 49]. Our study suggests that insomnia symptoms and self-efficacy should be considered as two important factors to be targeted to improve compliance with CPAP. Thus, it is necessary to conduct studies that provide evidence that interventions that reduce insomnia symptoms and improve self-efficacy will increase CPAP compliance and reduce morbidity/mortality associated with OSA.
